# Current Strategies for Microbubble-Based Thrombus Targeting: Activation-Specific Epitopes and Small Molecular Ligands

**DOI:** 10.3389/fbioe.2021.699450

**Published:** 2021-07-16

**Authors:** Zhaojian Wang, Huaigu Huang, Yuexin Chen, Yuehong Zheng

**Affiliations:** Department of Vascular Surgery, Peking Union Medical College Hospital, Beijing, China

**Keywords:** microbubbles, thrombosis, activation-specific probe, supramolecular carriers, small molecular ligand

## Abstract

Microbubbles with enhanced ultrasound represent a potentially potent evolution to the administration of a free drug in the treatment of thrombotic diseases. Conformational and expressional changes of several thrombotic biological components during active coagulation provide epitopes that allow site-specific delivery of microbubble-based agents to the thrombus for theranostic purpose. Through the interaction with these epitopes, emerging high-affinity small molecular ligands are able to selectively target the thrombi with tremendous advantages over traditional antibody-based strategy. In this mini-review, we summarize recent novel strategies for microbubble-based targeting of thrombus through epitopes located at activated platelets and fibrin. We also discuss the challenges of current targeting modalities and supramolecular carrier systems for their translational use in thrombotic pathologies.

## Introduction

Cardiovascular disease (CVD) remains the top cause of death worldwide and accounts for more than 40% of deaths in China (Liu et al., 2019). More than half of these deaths share the common pathogenesis pertaining to life-threatening thromboembolism that eventually leads to ischemic heart disease, ischemic stroke, and venous thromboembolism. The recognized significance of thrombosis in the pathogenesis of CVD has led to a great clinical demand for thrombus-specific agents that aid in the targeted delivery of imaging probes and therapeutics (Lanza et al., 2019; Zenych et al., 2020). To treat these severe thromboembolic diseases, fibrinolytic agents such as plasmin and plasminogen activators were developed and administered in patients through a systemic approach. However, there remain significant deficiencies in the current fibrinolytic therapy: low regional drug concentration, rapid drug elimination, unsatisfactory thrombolytic efficacy, and increased risk of bleeding (Zenych et al., 2020). Recent research studies have highlighted the success of emerging supramolecular drug delivery systems (DDSs) for their potential theranostic application in a variety of CVDs (Zhang et al., 2017; Bonnard et al., 2019; Disharoon et al., 2019; Yu et al., 2020). Several supramolecular platforms, such as lipid nanoparticles, polymeric nanocarriers, ultrasound-responsive microbubbles, and inorganic nanoparticles, may provide an alternative for site-specific diagnosis and treatment of CVD. Among them, ultrasound-responsive microbubbles hold a promising outlook for both detection and treatment of thrombosis. Microbubbles are spheroidal vesicles with a typical diameter of 1–8 μm (Disharoon et al., 2019). The upper limit of size is necessary and important for microbubbles to pass the human capillary (Stride, 2009). The coating material of microbubbles can be lipids, polymers, or denatured proteins (Zenych et al., 2020). Insoluble filling gas, like perfluorocarbon or a mixture of several gases, gives rise to the high compressibility and ultrasound-responsive property of microbubbles (Stride, 2009). Ultrasound-responsive microbubbles prevail in comparison with other supramolecular modalities because of the following reasons: (i) encapsulation of drug retards systemic deactivation (Kandadai et al., 2015); (ii) selective drug delivery reduces the risk of bleeding (Wang et al., 2016); (iii) microbubbles as functionalized contrast agents allow a more precise diagnosis and early detection of the thrombus (Dayton and Rychak, 2007; Li et al., 2019); (iv) spatiotemporal controlled drug delivery is achieved *via* the stimulation of ultrasound (Chowdhury et al., 2020); (v) combination with the ultrasound empowers higher thrombolytic efficacy (Laing et al., 2012; Lu et al., 2016); and (vi) real-time effect of the thrombolytic therapy can be visualized (Wang et al., 2012).

Enabling microbubbles with the capability of active targeting allows increased accumulation of drug at the site of thrombus and potentially enhances the penetration of therapeutic agents into the thrombus (Bonnard et al., 2014; Wang et al., 2020). High affinity and specificity are both desirable features when selecting an ideal targeting moiety for the DDS constructs. Here, we review recent strategies for the active targeting of thrombus in microbubble-based supramolecular DDSs that are promising for improving clinical efficacy and safety of diagnosis and treatment of thrombosis. We also discuss the rationale behind the choice of activation-specific epitopes and review the challenges of current targeting moieties for the theranostic applications in thrombotic pathologies.

## Targeting Rationale

An ideal target should be both unique in conformation and adequate in number in order to improve specificity and sensitivity. The process of thrombosis provides a number of promising targets for the design of thrombus-specific ultrasound-responsive multifunctional microbubbles. Sequential steps of arterial thrombosis (Mackman et al., 2020) (often resulting from atherosclerotic plaque rupture) are summarized as the following: (i) Vascular injury exposes subendothelial collagen and tissue factors (TFs), initiating the coagulation cascade. (ii) Collagen binds indirectly *via* von Willebrand factors (vWFs) and directly *via* membrane receptors to the platelets, inducing adhesion and activation of platelets. (iii) Mediators with cellular and plasmatic origin trigger further activation of platelets whose signals are reinforced *via* a positive feedback loop. (iv) Incremental expression of *P*-selectin on the platelets and injured endothelia promotes coagulation with adhered leukocytes. (v) Glycoprotein (GP) IIb/IIIa (integrin αIIbβ3) receptors are activated and mediate the aggregation of platelets through binding with fibrinogen and other ligands. (vi) Thrombin triggered by coagulation cascade converts fibrinogen to fibrin on the surface of platelet and activates factor XIII that crosslinks and stabilizes fibrin, resulting in the formation of thrombus. In venous thrombosis, hypoxia and inflammatory stimuli induced the coagulation cascade under low shear stress *via* the activation and regional release of relevant transcriptional factors [hypoxia-inducible factor 1 (HIF-1) and early growth response 1 (EGR-1)] and TFs (Bovill and van der Vliet, 2011).

**FIGURE 1:**
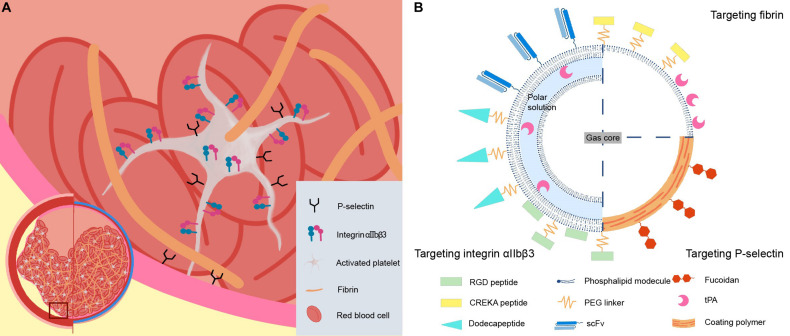
Schematic representations of **(A)** arterial and venous thrombus, candidate-targeting epitopes, and **(B)** microbubble-based thrombus-targeting strategies.

Among these steps, activated platelets and fibrin serve as two unique and attractive ligand-binding sites for targeted microbubbles. Activated platelet, as a homing platform demonstrates great potentials in molecular imaging and targeted drug delivery. Expressional and conformational changes of several surface proteins specifically located on the activated platelets provide structural prerequisites for targeted supramolecular DDS of the microbubble. In addition, the platelet-specific binding of the ligand is conditional, leading to the discrimination of platelet status that only activated platelets can be targeted, achieving high selectivity. Fibrin is not only a key acellular component of the thrombi but also deposits at the site of tissue injury and tumor invasion. Fibrin is present at high concentration in both arterial and venous thrombi, whereas it hardly exists in normal circulation, resulting in potentially high sensitivity and specificity for targeted constructs. Differences in the clotting process between the arterial and venous thrombi give rise to their distinctive features in thrombotic composition. Fibrin (43.3%), platelets (31.3%), and red blood cells (RBCs) (17.1%) are the major components of arterial thrombi. In contrast, the total content of RBCs in venous thrombi (63.4%) is significantly greater while the portion of platelet (0.4%) drastically decreased in comparison with arterial thrombi (Chernysh et al., 2020). However, both arterial and venous thrombi are rich in fibrin. Naturally, the composition of thrombi is an important factor in terms of choosing appropriate targeting candidates. In consideration of both abundance and uniqueness, integrin αIIbβ3, *P*-selectin on the activated platelets, and acellular component fibrin seem to be desirable targeting epitopes for activation-specific delivery of theranostic agents (Figure 1A).

## Targeting Integrin αIIbβ3

Integrin αIIbβ3, also known as GP IIb/IIIa receptor, is a heterodimeric transmembrane receptor that mediates aggregation of platelets through binding with circulating fibrinogen when activated (Cox et al., 2010). Integrin αIIbβ3 is platelet-specific (Phillips et al., 1991) and is the most abundant subtype of integrin found on the platelet (70,000–90,000 copies per platelet) (Zhou et al., 2011; Coller, 2015), making it a promising target for the thrombus-specific design of DDS. Under normal hemodynamic conditions, inactivated integrin αIIbβ3 is in a compact conformation in which the heads of α-subunit and β-subunit are bent and stabilized by clasps in the cytoplasmic tails, resulting in low affinity to ligands (Cox et al., 2010). Upon activation by intracellular signals or extracellular stimuli, it undergoes reorganization of the residues of amino acids, resulting in a swing-out motion of the head domains into a fully extended high-affinity state (Coller, 2015).

The restricted distribution and physiological tunability in ligand binding affinity of integrin αIIbβ3 lie in the foundation for higher specificity of the certain ligand, minimizing the off-target effects. Two ligand-memetic strategies have been used to target integrin αIIbβ3: the amino acid arginine, glycine, and aspartate (Arg-Gly-Asp) arginylglycylaspartic acid (RGD)-binding motifs and dodecapeptide adapted from fibrinogen gamma-chain C-terminal sequence. Bioconjugates with RGD motif are frequently taken in the design of targeting microbubbles, either absorbed onto the microbubble surface or covalently grafted *via* polyethylene glycol (PEG) linkers (Mu et al., 2009; Figure 1B). The mechanism of binding follows electrostatic rules that basic arginine residue interacts with the acidic Asp224 on a β-propeller region of αIIb subunit, and the aspartic acid carboxyl oxygen interacts with the metal ion-dependent adhesion site (MIDAS) magnesium (Mg^2+^) of β3 I domain (Coller, 2015). Less commonly, the dodecapeptide (HHLGGAKQAGDV) linked to liposome *via* PEGylation was also utilized for active targeting (Absar et al., 2013), based on coordination of carboxyl groups [(Asp) and valine (Val)] and metal ions [(Mg^2+^) and calcium (Ca^2+^)] at MIDAS and ADMIDAS (adjacent to MIDAS) region of β3 subunit (Absar et al., 2013; Coller, 2015). Fibrinogen gamma-chain dodecapeptide seems to have better binding specificity than RGD-based biomimetic ligands in terms of the cross-reactivity with other integrin receptors. Several modifications have been proposed to further enhance the affinity and specificity of RGD-based bioconjugates to integrin αIIbβ3. Forming more thermodynamically favorable closed structures, *via* the disulfide bonds between cysteine residues flanking the RGD motif, has been shown to not only increase the affinity but also potentially promote the ability to tissue penetration (Huang et al., 2008). Alternating amino acid residues near the RGD motif and synergy site distant to RGD motif present an attractive outlook in further refinement of targeting specificity toward certain types of integrin (Rubtsov et al., 2016). The potential application of RGD-modified microbubbles is not restricted in the detection and treatment of thrombus (Zhang et al., 2018; Guan et al., 2020). In other diseases where activated platelets play a significant role, such as high-risk atherosclerotic plaques in the preclinical stage of acute CVD, the functionalized microbubble can be utilized as biomarkers and can be quantified by ultrasound molecular imaging for the surveillance of disease activity (Guo et al., 2015; Gao et al., 2021).

Several modalities of bioconjugate ligands have been explored to target integrin αIIbβ3 of activated platelets including antigen-binding fragment (Fab) (Alonso et al., 2007), single-chain variable fragment (scFv) (Wang et al., 2012, 2016), and small peptides (Hagisawa et al., 2013; Wu et al., 2013). Early antibody-based bioconjugate Abciximab is not further investigated due to its limitations in cross-reactivity with other integrins and non-selective binding regardless of the platelet status (Coller, 1999; Schwarz et al., 2002). In general, small molecular ligand memetics, such as peptides and scFv, are superior as targeting moieties because of their relatively low immunogenicity, simple structure, convenient synthesis, easy modification, and low cost (Wang et al., 2012). Notably, these targeting moieties allow enrichment of fibrinolytic agents at the thrombus site while reducing systemic concentration at the same time (Wang et al., 2016). However, safety concerns have been raised over the induced conformational change and outside-in signaling with integrin αIIbβ3 by these biomimetic ligands, causing paradoxical bioeffects including thrombotic propagation especially when the concentration of systemic ligand remains low (Ahrens and Peter, 2008). To address this important issue, an activation-specific scFv, obtained from phage-display libraries, was found to interact with receptors specific to activated platelet without inducing conformational changes or outside-in signals, minimizing the unwanted physiological effects that have raised safety concerns (Schwarz et al., 2006). Sortase-mediated bioconjugation or biotin-streptavidin interaction was employed to attach the scFv to desired supramolecular carriers with a binding capacity of the scFv to activated platelets that retained (Wang et al., 2012; Kim et al., 2015; Figure 1B). Mutagenesis studies and computer modeling indicate that such conformation-specific binding is mediated by RGD motifs in the heavy-chain complementary-determining region 3 (HCDR3) of scFv that forms an exceptionally extended loop (Schwarz et al., 2006). The sequence and unique structure highlighted by the HCDR3 region may inspire designs for the future development of activation-specific low molecular probes with better safety profiles based on the information of this template.

## Targeting *P*-Selectin

*P*-selectin is a cell adhesion molecule that is rapidly transferred to the surface of activated platelets and injured vascular endothelium (André, 2004). Cytoplasm-integrated *P*-selectin belongs to the type I membrane protein with an N-terminal carbohydrate-recognition motif, C-type lectin, an epidermal growth factor (EGF)-like motif, a series of nine consensus repeats, a transmembrane domain, and the C-terminal cytoplasmic tail (André, 2004). The main ligand of *P*-selectin is *P*-selectin glycoprotein ligand 1 (PSGL-1), the recognition of which is based on tetrasaccharide sialyl Lewis X (sLex) and three sulfated tyrosine residues in the protein backbone of PSGL-1 (Bachelet et al., 2009). As an adhesion molecule, *P*-selectin promotes the interaction of platelets, endothelia, and leukocytes in inflammatory and hemostatic cellular processes and thus represents an important molecular target in acute and chronic CVDs (Rouzet et al., 2011). *P*-selectin is minimally expressed on the resting membrane prior to activation (Thomas and Storey, 2015). Upon activation, *P*-selectin is released to the cytoplasm *via* cytoplasmic granules with a density of around 10,000 molecules per activated platelet (André, 2004), mediating rolling of leukocytes on activated endothelium and formation of platelet-leukocyte aggregates with PSGL-1, causing growth and stabilization of the thrombus (André, 2004). The distinct differences in the expressional pattern of *P*-selectin on the cytoplasm in response to activation provide the basis for activation-specific ligand recognition.

A number of synthetic sLex memetics, sulfated oligosaccharides, and polysaccharides have been identified to have interactions with *P*-selectin. Among them, fucoidan, a sulfated L-fucose-based polymer extracted from seaweed, excels for its easy accessibility, reliable biosafety (Chauvierre et al., 2019), and higher affinity (dissociation constant, *K*_*D*_ = 1.2 nM) (Bachelet et al., 2009) for immobilized *P*-selectin *in vitro* compared to its natural ligand (*K*_*D*_ = 320 nM) (Mehta et al., 1998; Figure 1B). Recently, fucoidan-coated polymeric microbubbles were developed for the targeting of activated platelets in the thrombotic abdominal aortic aneurysm (AAA) (Li et al., 2017). To construct these microbubbles, coating material, in which fucoidan and dextran are covalently linked to, was formed by means of redox radical emulsion polymerization or anionic emulsion polymerization of isobutyl cyanoacrylate (IBCA) in an aqueous solution (Lira et al., 2011; Li et al., 2017, 2019). After the construction, flow cytometry and flow chamber experiments confirmed the binding specificity of microbubbles in terms of selectin subtypes (*P*-selectin but not *L*-/*E*-selectins) and platelet status (activated platelets but not quiescent platelets) (Li et al., 2017). *In vivo* administration of the microbubbles in a rat AAA model confirmed this differentiated binding to thrombotic AAA even at a high shear rate (Li et al., 2017). In addition, fucoidan-functionalized polysaccharide microbubbles have been developed in both venous and arterial models as a new ultrasound contrast agent or a new tool for single-photon emission computed tomography (SPECT) imaging in the molecular diagnosis of cardiovascular lesions (Itoh et al., 2014; Li et al., 2019). Interestingly, compared with radiolabeled fucoidan alone whose tissue uptake and retention are more localized in the thrombus itself, fucoidan-functionalized supramolecular DDS was reported to favor deeper tissue penetration to the injured vessel walls with the hypothesis that such microscale/nanoscale carriers possessed an improved hemodynamic behavior that, to some extent, mimicked the migration of leukocyte to the lesion (Bonnard et al., 2014).

Other moieties, such as small peptides (Modery et al., 2011) and constructs based on bio-orthogonal coupling chemistry (Wang et al., 2017), have been developed while each of these methods has its own limitations compared to polysaccharides. Mechanisms of the latter approach are based on the rapid and selective Diels-Alder reaction between *trans*-cyclooctene (TCO)-tagged *P*-selectin-specific antibodies and tetrazine-tagged microbubbles, which empowered the ultrasound imaging with a specific, rapid, and sensitive contrast agent (Wang et al., 2017). However, additional studies including pharmacokinetics and pharmacodynamics of the targeted moieties are required. Notably, the biosafety, accessibility, and cost of such constructs should also be considered.

## Targeting Fibrin

In the past, molecular targeting of fibrin was limited by the challenge of discerning the fibrin from fibrinogen, a component that shares 98% of its structure with fibrin and presents in high concentrations (2.5–3 mg/ml) in the blood (Standeven et al., 2005). Recently, specificity for fibrin over fibrinogen has been achieved by several molecular entities such as selective antibodies, small peptides, and tissue plasminogen activator (tPA) (Oliveira and Caravan, 2017).

As antibodies are restricted by their large size, immunogenicity, random orientation upon conjugation, and high cost, small peptides are equally desirable for fibrin-targeted strategies as previously mentioned in the integrin αIIbβ3 section. The Cys-Arg-Glu-Lys-Ala (CREKA) peptide, which was identified by *in vivo* screening of phage-display peptide libraries in tumor-bearing mouse mammary tumor virus-polyoma middle tumor-antigen (MMTV-PyMT) transgenic breast cancer mice initially designed for tumor homing (Hoffman et al., 2003), has become an attractive candidate among peptides that have been identified to bear high affinity toward fibrin. While no crystallographic information on the binding of CREKA to the fibrin-fibronectin complex is available, both *in vitro* and *in vivo* studies have confirmed the accumulation of CREKA-modified constructs at the fibrin deposition site evident by the colocalization of fluorescent markers and significantly intensified signals compared to the control (She et al., 2014; Lu et al., 2019). Such binding is highly selective as no accumulation of CREKA peptide was identified in the fibrinogen or fibronectin null mice (Simberg et al., 2007; Agemy et al., 2010). A combination of simulated annealing and molecular dynamics has revealed energy landscape and bioactive conformation of CREKA that involves a β-turn motif determined by ionized side chains of Arg, Glu, and Lys with a pattern of multiple interactions including salt bridge and hydrogen bonds (Zanuy et al., 2008). Moreover, the bioactive conformational profile of the peptide is found to be independent of the chemical environment, allowing conjugation of CREKA to other entities without significant alterations in the binding pattern (Zanuy et al., 2008). In fact, the sulfhydryl group of the single cysteine residue is not required in terms of binding activity and can be utilized for covalent coupling *via* a PEG linker with microbubbles (Simberg et al., 2007; Gormley et al., 2019; Figure 1B). Recently, modifications such as incorporation to the cyclic backbone (Zhang et al., 2019) and analogs with proteolysis-resistant residue (Zanuy et al., 2020) have been proposed to further enhance the efficacy and stability of CREKA-targeting peptide. Song et al. (2014) use CREKA peptides as targeting modules to specifically and effectively detect microthrombus with non-invasive imaging equipment, which allows for more sensitive and penetrating imaging techniques to subtle anatomical structures. By adding CREKA peptide to phase-transitional constructs, Zhong et al. (2019) realized simultaneous non-invasive detection and non-pharmaceutical phase-transitional thrombolysis, demonstrating the great potentials of such targeted supramolecular DDS in the diagnosis and treatment of acute CVD.

Another strategy to assemble a fibrin-targeting construct is to modify the surface of the construct with tPA, a fibrin-specific enzyme in physiologic fibrinolysis. Upon fibrin assembly, conformational changes in the αC domain of fibrin cause the exposure of α148–160 epitopes in its finger domain and γ312–324 epitopes in its kringle-2 (K2) domain, which are involved in enhanced plasminogen activation by tPA (Mosesson, 2005). Both sites are cryptic in fibrinogen but become exposed during fibrin assembly, ensuring the specificity of targeted binding. Affinities of tPA to these interactive sites are high, 2 nM for the finger domain and 33 nM for the K2 domain, respectively (Klegerman et al., 2008). Further, such high-affinity interactions are fully retained in the conjugated form of tPA to the echogenic liposomes (ELIPs), resulting in high sensitivity of the site-specific targeting (Klegerman et al., 2008). One of the most attractive characteristics of tPA bioconjugates is its combined targeting and therapeutic functions, which not only broadens the application spectrum of such constructs but also provides convenience in assembly. The targeting efficacy and thrombolytic efficacy of tPA-loaded ELIP have been confirmed in an *in vitro* clot model and *in vivo* rabbit aorta thrombus model (Tiukinhoy-Laing et al., 2007; Laing et al., 2011). In this study, the preparation of ELIP followed the sonication-lyophilization-rehydration method, and recombinant tPA was introduced at the initial rehydration of the lipid film. This method resulted in two types of incorporation of tPA: 70% were associated with the lipid bilayer, whereas 30% were fully encapsulated (Tiukinhoy-Laing et al., 2007; Figure 1B). This bifunctional supramolecular DDS can potentially lead to sensitive and selective delivery of tPA by effectively lengthening its half-life, avoiding systemic deactivation, and improving selectivity. Wang et al. (2020) reported the decrease of residual thrombus by 67.5% and higher drug penetration in microbubble-based tPA delivery system when compared to conventional systemic administration of tPA, highlighting the advantageous therapeutic efficacy in time-critical thrombolytic therapy.

## Conclusion and Challenges

In summary, we present a brief overview of the strategies available to implement the thrombus-specific targeting function of microbubbles. Compared to traditional antibody-mediated targeting strategies, emerging high-affinity small molecular biomimetic ligands for activation-specific targeting have become popular research and development objects with their unique advantages. Activation-dependent epitopes that are highly abundant and uniquely thrombus-specific during active coagulation are under extensive investigation for their promising outlook in the future of targeted supramolecular DDS, including integrin αIIbβ3, *P*-selectin, and fibrin αC domain. Despite the attractive prospects, there still exist challenges that may influence the theranostic application of the thrombus-specific small molecular targeting moieties.

(i) Thrombosis is a dynamic pathophysiological process, and the pathological composition of the thrombus evolves with the age of thrombi (Yusof et al., 2019). Such changes in composition result from the complicated interplay of coagulation factors, leukocytes, cytokines, protease, and other factors (Yusof et al., 2019). Epitopes of the thrombus may change along with their composition. For example, fibrin as an important epitope of thrombus would decrease, while collagen deposition would increase in the aging thrombus. Most of the studies available examined the targeting efficacy of ligands with thrombi of the acute phase (Wang et al., 2012, 2017; Li et al., 2019; Lu et al., 2019), while the efficacy in subacute or chronic thrombi remained unknown (Wu et al., 2013). Therefore, the influence of the age of thrombus on the targeting capability of ligands has to be systemically evaluated further. Alternatively, the variations in the binding of the targeting agent, reflected by the difference in contrast intensity, may serve as a differential index for the assessment of thrombus maturity in clinical settings.

(ii) Optimization of the targeting agents and carrier system is required for maximal sensitivity and specificity of supramolecular DDSs *in vivo*. The preparation procedures of functionalized microbubble agents are complex, and parameters including microbubble size, conjugation methods, loading amount of targeting agents, and the ratio of targeting and therapeutic components were reported to influence targeting and therapeutic efficacy of drug carriers (Mu et al., 2009; Shamloo et al., 2020; Wang et al., 2021). Recently, a heteromultivalent ligand decoration strategy that targets two different epitopes has been examined for their capability of active anchoring to thrombi (Günther et al., 2017; Maier et al., 2017; Pawlowski et al., 2017). Despite the promising outcomes *in vitro*, the superior binding effect of dual-targeting strategy over single-targeting strategy seemed to be not translatable *in vivo*. Continuous efforts are desirable to optimize parameters that collectively generate an ideal targeting modality in the future.
(iii) There exists a huge gap between scientific benchwork and clinical practice. Despite the vigorous research activity in the pertinent field with the architecture of microbubble-based DDSs becoming increasingly complex and diverse, the progress of clinical translation remains slow. Logistic challenges of this targeted supramolecular DDSs lying ahead include product reproducibility, storage stability, off-target biodistribution, and biosafety issues (Wang et al., 2012; Pawlowski et al., 2017). In an effort to overcome these challenges, the first thrombus-specific supramolecular DDS tested in human was estimated to be the simple combination of approved commercial fibrinolytic drugs and microbubbles with a relatively predictable risk and stable safety profile (Zenych et al., 2020). More sophisticated variations of microbubble-based DDSs encompassing stimuli-responsive control of drug release and dual targeting or dual therapeutic agents may be approved for tests in human building on the success of the former simple version.

In-depth investigations into these challenges will significantly advance the strategies for the rational design of microbubble-based thrombus-targeting moieties, thereby substantially empowering the supramolecular DDS as a potential game changer in medicine for their theranostic use in a number of thrombotic pathologies.

## Author Contributions

ZW, HH, YC, and YZ conceptualized and planned the manuscript. ZW created the figures and wrote the manuscript. HH, YC, and YZ made critical revisions to the manuscript. All authors have approved the manuscript.

## Conflict of Interest

The authors declare that the research was conducted in the absence of any commercial or financial relationships that could be construed as a potential conflict of interest. The reviewer YZ declared a shared affiliation, with no collaboration, with the authors ZW, HH, YC, and YZ to the handling editor at the time of the review.
